# Modeling of Rate-Independent and Symmetric Hysteresis Based on Madelung’s Rules

**DOI:** 10.3390/s19020352

**Published:** 2019-01-16

**Authors:** Kairui Cao, Rui Li

**Affiliations:** School of Astronautics, Harbin Institute of Technology, Harbin 150001, China; rlihit@hotmail.com

**Keywords:** hysteresis, Prandtl-Ishlinskii model, Madelung’s rule, turning point

## Abstract

Hysteresis is a kind of nonlinearity with memory, which is usually unwanted in practice. Many phenomenological models have been proposed to describe the observed hysteresis. For instance, the Prandtl-Ishlinskii (PI) model, which consists of several backlash operators, is the most widely used. On the other hand, the well-known Madelung’s rules are always used to validate hysteresis models. It is worth pointing out that the PI model obeys Madelung’s rules. In this paper, instead of considering these rules as criteria, we propose a modeling method for symmetric hysteresis by directly constructing the trajectory based on Madelung’s rules. In the proposed method, turning points are recorded and wiped out according to the input value. After the implementation of the recording and wiping-out mechanisms, the curve which the current trajectory moves along can be determined and then the trajectory can be described. Furthermore, the relationship between the proposed method and the PI model is also investigated. The effectiveness of the presented method is validated by simulation and experimental results.

## 1. Introduction

Hysteresis is the phenomenon that can be found in a wide variety of smart materials [1,2,3], such as piezoceramics [4,5,6], magnetostrictive materials [7], and shape memory alloys [8]. It brings considerable problems to the application of smart materials because the output of the system actuated by these materials cannot be predicted without the knowledge of the hysteresis behavior of the system. Generally, it is hard to give an accurate and general model through basic physical principles of a material. To simulate the observed hysteresis behavior, many phenomenological models have been developed in the literature, e.g., Bouc-Wen [9,10,11,12], Duhem, Preisach model [1], Maxwell [4], and Prandtl-Ishlinskii (PI) models [13]. 

The above phenomenological models can further be classified into two categories: differential-based models and operator-based models. The Bouc-Wen and Duhem models are differential-based models since they contain the derivative of the output in the model. Compared with the Duhem model, the Bouc-Wen model is more popular since it has only a few parameters to be identified. For this reason, it has been widely used in precision control systems actuated by piezoelectric ceramics [10,11]. However, it was shown in Reference [12] that the Bouc–Wen model is inferior to the Preisach model in the trajectory prediction of piezoelectric actuators (PEAs). The inverse of a hysteresis model is also important since it can be used as a feedforward controller to reduce the hysteretic effect. Unfortunately, the construction of the inverse for the Bouc–Wen model is a difficult task since it cannot be inversed analytically [3]. In Reference [9], a Bouc–Wen least square support vector machine was proposed for hysteresis compensation without the need to model the inverse. A multiplicative inverse structure was proposed in Reference [10] to compensate the hysteresis modeled by the Bouc–Wen model. The Preisach model, Maxwell model, and PI model all belong to operator-based models. The main idea of operator-based models is to use the weighted sum of several simple motions to describe complex movements. For example, the simple relay hysteron is the basic operator in the Preisach model. Generally, the number of adopted operators influences the accuracy of the Preisach model. The accuracy grows with the number of operators. However, the computation time also increases. It is not convenient to use the Preisach model in real-time applications since it cannot be inversed analytically. Some strategies were proposed to overcome these disadvantages of the Preisach model, such as the inverse multiplicative technique [14] and the recursive approach [15]. As for the Maxwell model, the basic operator is the elasto-slide element, which consists of a massless linear spring and a massless block that suffers from Coulomb friction. To give a physical insight into PEAs, the elasto-slide element was replaced with the resistive capacitance element in Reference [4]. Among the operator-based models, the PI model is the most widely used in practice [10,13]. It is a weighted superposition of several backlash operators. One of the advantages of the PI model lies in the fact that its inverse exists and is also of the PI type. The parameters of the inverse PI model can be derived from those of the PI model, and this property facilitates the design of feedforward controllers. It should be emphasized that the above mentioned phenomenological models are only good at describing rate-independent and symmetric hysteresis.

However, the observed hysteresis is always rate-dependent or asymmetric, or both of them. A rate-dependent circuit model was proposed in Reference [16], where the creep phenomenon was also considered. Note that this model has an equivalent hardware circuit. In References [17,18], the rate-dependent PI model was developed by dynamically changing its threshold values according to the input rate. In Reference [19], the Bouc–Wen model cascaded with a linear dynamic model was used to describe rate-dependent hysteresis. Based on the classical PI model, many modified forms have been presented to describe asymmetric hysteresis [13,20,21] and rate-dependent hysteresis [22,23,24]. In essence, the core of these models is still the classical PI model. Therefore, the investigation for rate-independent and symmetric hysteresis models is important, since they are the basis of describing rate-dependent and asymmetric hysteresis.

On the other hand, German physicist Madelung presented three rules based on his observation of the hysteresis phenomenon in the early 20th century. These rules are always used to validate hysteresis models [25], such as the Preisach model and the PI model [13]. In this paper, instead of considering Madelung’s rules as criteria, we propose a modeling method for the rate-independent and symmetric hysteresis based on these rules. In our method, turning points are recorded as the record of movement history and then wiped out according to the input value. After the implementation of the recording and wiping-out mechanisms, a key turning point named as the current turning point (CTP) is determined. Then the trajectory of the symmetric hysteresis can be described by this CTP. The contributions of this paper are listed as follows:

- We propose a modeling method to describe the symmetric hysteresis by directly constructing its trajectory based on Madelung’s rules rather than considering these rules as criteria. Furthermore, this method is translated into an algorithm that can be run by digital processors.
- The relationship between the proposed method and the PI model is investigated.

The remainder of this paper is organized as follows: In Section 2, the proposed hysteresis modeling method is explained in detail. Simulations and experiments are conducted in Section 3 to show the effectiveness of this method. At last, some discussions and conclusions are drawn in Section 4.

## 2. Trajectory Construction Method

### 2.1. Madelung’s Rules and Their Applications in Trajectory Construction

**Figure 1:**
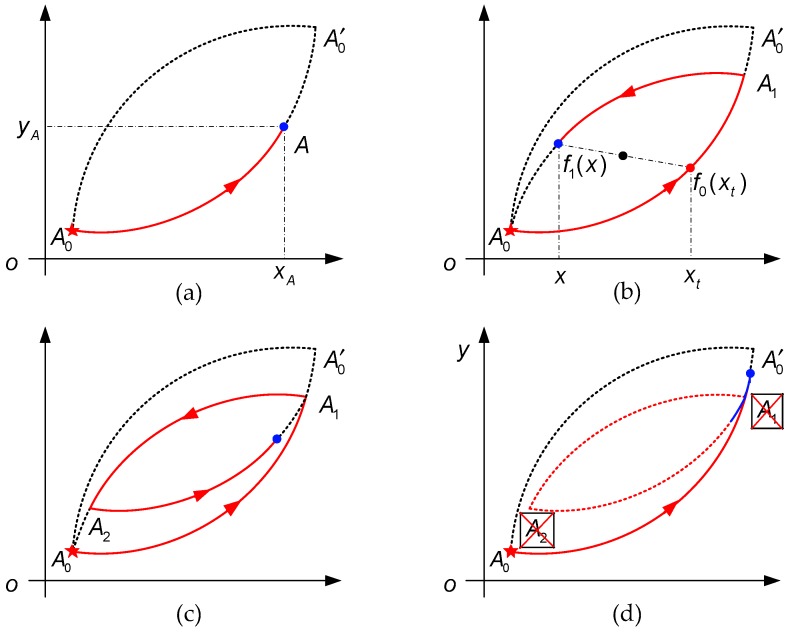
Schematic illustration of Madelung’s rules. (**a**) Trajectory on the major ascending curve $A_{0}A_{0}^{\prime }$; (**b**) Trajectory on $A_{1}A_{0}$; (**c**) Trajectory on $A_{2}A_{1}$; (**d**) Transfer of the trajectory from $A_{2}A_{1}$ to $A_{0}A_{0}^{\prime }$.

In this subsection, the definition of turning point is first given. A turning point is a point on the trajectory where the input changes its direction, for instance, point $A_{0}$ and point $A_{1}$ as shown in Figure 1a,b, respectively. For point A on the trajectory, we use $x_{A}$ and $y_{A}$ to represent its x− and y− coordinates, respectively (see Figure 1a). Before introducing our method, we list Madelung’s rules as follows [13,25]:

1. Any trajectory starting from a turning point is uniquely determined by the coordinates of this point. For example, the turning point $A_{1}$ in Figure 1b is the starting point of curve $A_{1}A_{0}$, as we will see in the next that the function of $A_{1}A_{0}$ can be uniquely described by $x_{A_{1}}$ and $y_{A_{1}}$.
2. If any point $A_{2}$ on the trajectory, as shown in Figure 1c, becomes a new turning point, then the trajectory leads back to the previous turning point $A_{1}$. In other words, any hysteresis loop is closed.
3. If the trajectory moving along curve $A_{2}A_{1}$ is continued beyond $A_{1}$, then it coincides with the continuation of curve $A_{0}A_{0}^{\prime }$ as if hysteresis loop $A_{1}A_{2}-A_{2}A_{1}$ did not exist, as shown in Figure 1d.
  In addition to the above three rules, a fourth rule can be given for symmetric hysteresis.
4. The hysteresis loops of symmetric hysteresis are centrally symmetric.

In our method, it is assumed that the starting point of the hysteretic trajectory is point $A_{0}$ which corresponds to the minimum input value, as shown in Figure 1a. Starting from point $A_{0}$, the trajectory moves along the major ascending curve $A_{0}A_{0}^{\prime }$. Note that the function of $A_{0}A_{0}^{\prime }$ is $f_{0}(x)$, where $x\in D_{f_{0}}=[A_{0},\;A_{0}^{\prime }]$ and $D_{f_{0}}$ is the domain of $f_{0}(x)$. According to Rule 2, the trajectory leads back to $A_{0}$ along $A_{1}A_{0}$ after it is reversed at point $A_{1}$, where $x_{A_{1}}\in (x_{A_{0}},\;x_{A_{0}^{\prime }}]$ (see Figure 1b). We can see from Rule 4 that $A_{1}A_{0}$ and $A_{0}A_{1}$ are centrally symmetric as illustrated in Figure 1b, and, thus, point $\left(x,f_{1}(x)\right)$ on $A_{1}A_{0}$ and its corresponding point $\left(x_{t},f_{0}(x_{t})\right)$ on $A_{0}A_{1}$ has the following relationship:

(1) $$\left\{ \begin{array}{l} \frac{x+x_{t}}{2}=\frac{x_{A_{0}}+x_{A_{0}^{\prime }}}{2}\\ \frac{f_{1}(x)+f_{0}(x_{t})}{2}=\frac{f_{0}(x_{A_{0}})+f_{0}(x_{A_{0}^{\prime }})}{2} \end{array}\right.$$

where $\left(x_{A_{0}}+x_{A_{0}^{\prime }})/2,\;(f_{0}(x_{A_{0}})+f_{0}(x_{A_{0}^{\prime }}))/2\right)$ is the central point of hysteresis loop $A_{1}A_{2}-A_{2}A_{1}$. Removing $x_{t}$ in Equation (1), the function of $A_{1}A_{0}$ can be expressed by

(2) $$f_{1}(x)=f_{0}(x_{A_{0}})+f_{0}(x_{A_{1}})-f_{0}(x_{A_{0}}+x_{A_{1}}-x),\;x\in D_{f_{1}}=(x_{A_{0}},\;x_{A_{1}}).$$

If point $A_{2}$ on $A_{1}A_{0}$ becomes a new turning point, the new curve $A_{2}A_{1}$ can be described by

(3) $$f_{2}(x)=f_{1}(x_{A_{1}})+f_{1}(x_{A_{2}})-f_{1}(x_{A_{1}}+x_{A_{2}}-x),\;x\in D_{f_{2}}=(x_{A_{2}},\;x_{A_{1}}).$$

At this time, the trajectory moves along curve $A_{2}A_{1}$ as illustrated in Figure 1c. If the trajectory is continued beyond the former turning point $A_{1}$, the trajectory will then move onto curve $A_{0}A_{0}^{\prime }$ as if hysteresis loop $A_{1}A_{2}-A_{2}A_{1}$ did not exist at all (see Rule 3), which means $A_{1}$ and $A_{2}$ can be wiped out. Otherwise, the trajectory should be reversed at turning point $A_{3}$ which belongs to $A_{2}A_{1}$, and the function of the new curve $A_{3}A_{2}$ can be written as

(4) $$f_{3}(x)=f_{2}(x_{A_{2}})+f_{2}(x_{A_{3}})-f_{2}(x_{A_{2}}+x_{A_{3}}-x),\;x\in D_{f_{3}}=(x_{A_{2}},\;x_{A_{3}}).$$

Similarly, we can construct the following trajectory for any input signal. Without loss of generality, when turning points $A_{0},\;A_{1},\;\cdots ,\;A_{m}$ are generated in sequence, the function of curve $A_{k}A_{k-1}$ can be expressed by

(5) $$f_{k}(x)=f_{k-1}(x_{A_{k-1}})+f_{k-1}(x_{A_{k}})-f_{k-1}(x_{A_{k-1}}+x_{A_{k}}-x),\;\{\begin{array}{c} x\in D_{f_{k}}=(x_{A_{k-1}},\;x_{A_{k}})\;for\;k\;is\;even\\ x\in D_{f_{k}}=(x_{A_{k}},\;x_{A_{k-1}})\;for\;k\;is\;odd \end{array}$$

where k=1, 2, ⋯, m. It is noted that Equation (5) is not suitable for real-time applications since it is an iterative equation. In fact, Equation (5) can be simplified as follows:

(6) $$f_{k}(x)=\{\begin{array}{c} f_{k-1}(x_{A_{k}})-f_{0}(x_{A_{0}})+f_{0}(x_{A_{0}}+x_{A_{k}}-x),\;x\in D_{f_{k}}=(x_{A_{k-1}},\;x_{A_{k}})\;for\;k\;is\;even\\ f_{k-1}(x_{A_{k}})+f_{0}(x_{A_{0}})-f_{0}(x_{A_{0}}-x_{A_{k}}+x),\;x\in D_{f_{k}}=(x_{A_{k}},\;x_{A_{k-1}})\;for\;k\;is\;odd \end{array}$$

where $f_{k-1}(x_{A_{k}})$ is the y-coordinate of point $A_{k}$. Note that there are three terms in Equation (6) for each value of k, the first term is $f_{k-1}(x_{A_{k}})$ and the other two terms can be computed by $f_{0}(x)$. This means that curve $A_{k}A_{k-1}$ can be considered to be determined by the coordinates of point $A_{k}$ and, thus, Rule 1 is validated. According to Rule 3, the trajectory is transferred from $A_{k}A_{k-1}$ to $A_{k-2}A_{k-3}$ (k=3, 4, ⋯, m) once point $A_{k-1}$ is surpassed.

Until now, we have shown how to use Madelung’s rules to describe the trajectory of symmetric hysteresis. Next, we will translate this method into a computer algorithm. Before we proceed, some observations are made as follows:

- As the recorders of the movement history, all the turning points should be identified and recorded.
- If the trajectory surpasses previous turning point $A_{k-1}$, it will be transferred to the previous curve $A_{k-2}A_{k-3}$. Point $A_{k-1}$ and its previous point $A_{k}$ should be wiped out since they are of no use to describe the future trajectory.
- The domain range of the curve described by Equation (6) is decreasing with k, which means that
  (7) $$D_{f_{k}}\subset D_{f_{k-1}}\subset \cdots \subset D_{f_{1}}\subset D_{f_{0}}$$
- The trajectory at any time instant must be on one of the curves described by Equation (6), namely the current curve. If the current curve is determined, the trajectory can then be described. In fact, this curve can be determined by finding the minimum domain that contains the input value x among the existing curves. Mathematically, we have
  (8) $$D_{c}=\underset{\sigma (D_{f})}{\min}\{x\in D_{f},\;D_{f}\in D_{\mathrm{all}}\}$$
  where $D_{c}$ is the domain of the current curve, $\sigma (D_{f})$ represents the length of $D_{f}$, and $D_{\mathrm{all}}=\{D_{f_{0}},\;D_{f_{1}},\;\cdots ,\;D_{f_{m}}\}$.
- If k is odd, the curve described by Equation (6) is a descending curve, and it is an ascending curve when k is even.

### 2.2. Algorithm and Complex Analysis

**Figure 2:**
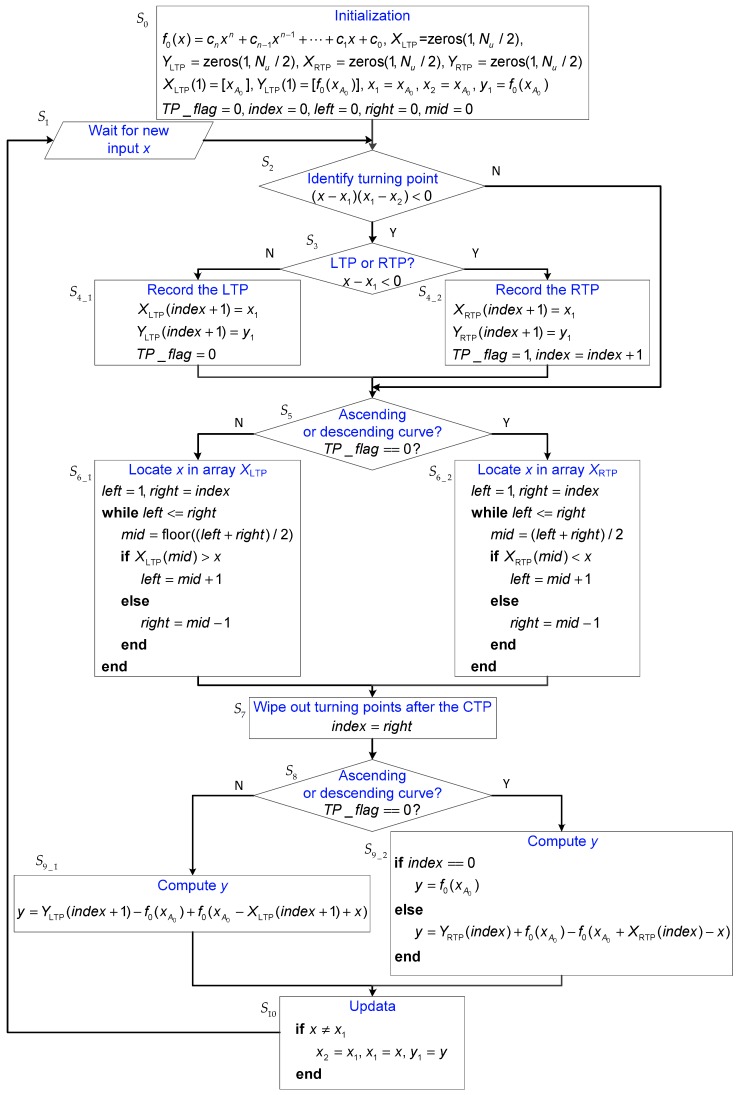
Flowchart of the trajectory construction method.

Based on the above analysis, it is obvious that the trajectory at any time instant must be on the current curve. The starting point of this curve is defined to be the current turning point (CTP). Once the CTP is determined, the trajectory can be described by Equation (6) according to Rule 1. The flowchart of the algorithm is shown in Figure 2, where each block is labeled to facilitate the presentation.

At the beginning, the initialization is conducted in $S_{0}$. The function of the major ascending curve is $f_{0}(x)$, and it is described by the following polynomial

(9) $$f_{0}(x)=c_{n}x^{n}+c_{n-1}x^{n-1}+\cdots c_{1}x+c_{0}$$

where $c_{i}$ (i=0, 1, ⋯, n) is the coefficient and n is the degree of the polynomial. In the algorithm, TPs are further classified into two types: left turning points (LTPs) and right turning points (RTPs). For instance, $A_{0},\;A_{2},\;A_{4},\;\cdots$ are LTPs and $A_{1},\;A_{3},\;A_{5},\;\cdots$ are RTPs. Array $X_{\mathrm{LTP}}$ and array $Y_{\mathrm{LTP}}$ are used to record the x− and y− coordinates of LTPs, respectively. Similarly, $X_{\mathrm{RTP}}$ and $Y_{\mathrm{RTP}}$ are used to record the coordinates of RTPs. Auxiliary variables $x_{1}$, $y_{1}$, and $x_{2}$ are utilized to identify turning points. The Boolean variable TP_flag is the indicator for the type of the latest turning point. For instance, TP_flag=0 implies that the latest turning point is an LTP and the trajectory moves along some ascending curve. The integer variable index represents the position of the CTP in turning point arrays. Furthermore, temporary variables left, right, and mid are used in the binary search method. In $S_{1}$, the new input value x is read into the program. The remainder of the algorithm can mainly be divided into three parts:

The first part consists of $S_{2}$, $S_{3}$, $S_{4\_1}$, and $S_{4\_2}$. The first step aims to identify turning points. To identify whether the previous point $\left(x_{1},\;y_{1}\right)$ is a turning point, the following inequality is utilized.

(10) $$(x-x_{1})(x_{1}-x_{2})<0$$

where $x_{1}$ and $x_{2}$ are two input values previous to x. If Inequality (10) holds, point $\left(x_{1},\;y_{1}\right)$ is a turning point. Furthermore, it is an LTP if $x-x_{1}>0$ and it is an RTP if $x-x_{2}<0$. Next, this point is restored in either $S_{4\_1}$ or $S_{4\_2}$ according to its type. 

**Figure 3:**
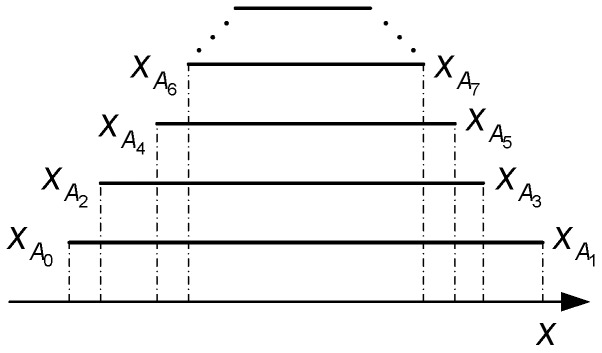
Domains of functions $f_{k}(x)$, k=1, 2, ⋯m.

The second part contains $S_{5}$, $S_{6\_1}$, $S_{6\_2}$, and $S_{7}$. The objective of this part is to determine the CTP and to wipe out the turning points after the CTP. As mentioned earlier, the current curve and the CTP can be determined by Equations (7) and (8). Herein, we will give simplified results. We can see from Equation (7) that $X_{\mathrm{LTP}}$ and $X_{\mathrm{RTP}}$ are naturally sorted in ascending order and descending order, respectively. To see the fact, the domains of the functions described by Equation (6) are plotted in Figure 3, where we have

(11) $$\left\{ \begin{array}{l} x_{A_{0}}<x_{A_{2}}<x_{A_{4}}<x_{A_{6}}<\cdots \\ x_{A_{1}}>x_{A_{3}}>x_{A_{5}}>x_{A_{7}}>\cdots \end{array}\right.$$

If TP_flag=0, the latest turning point $A_{m}$ is an LTP and the current curve is an ascending curve. Furthermore, we have $x_{A_{0}}<x_{A_{1}}<\cdots <x_{A_{m}}<x$, and, thus, Expression (8) can be simplified as follows:

(12) $$D_{f}=\left\{ \begin{array}{l} D_{f_{m}},\;x<x_{A_{m-1}}\\ D_{f_{k}},\;x_{A_{k}}\leq x<x_{A_{k-2}},\;k=3,\;5,\;\cdots ,m-1\\ D_{f_{0}},\;x_{A_{1}}\leq x\leq x_{A_{0}^{\prime }} \end{array}\right.$$

On the contrary, if TP_flag=1, the turning point $A_{m}$ is an RTP and Expression (8) can be rewritten as

(13) $$D_{f}=\left\{ \begin{array}{l} D_{f_{m}},\;x_{A_{m-1}}<x\\ D_{f_{k}},\;x_{A_{k-2}}\leq x<x_{A_{k}},\;k=\;4,\;6,\;\cdots ,m-1\\ D_{f_{0}},\;x_{A_{0}}\leq x\leq x_{A_{2}} \end{array}\right.$$

Apparently, the CTP can be determined by locating the position of x in $X_{\mathrm{LTP}}$ or $X_{\mathrm{RTP}}$. As shown in $S_{6\_1}$ and $S_{6\_2}$ of Figure 2, the binary search method is used to locate the position of x. After the “while” operation, the value of right is exactly the target position. Furthermore, all the turning points after the CTP are wiped out by setting index=right in $S_{7}$.

The third part comprises $S_{8}$, $S_{9\_1}$, $S_{9\_2}$, and $S_{10}$. In this part, the output y is computed using the CTP (see Equation (6)). In addition, auxiliary variables are updated only when $x\neq x_{1}$, since successive identical input values will influence the identification of turning points.

Owing to the wiping-out mechanism, the number of turning points cannot be increased infinitely. The maximum number of turning points equals to $N_{u}$, where $N_{u}$ is the number of different input values. For instance, a data acquisition system with a 12-bit digital-to-analog converter (DAC) has $N_{u}=2^{12}=4096$ different input values. On the other hand, since LTPs and RTPs are generated alternatively and eliminated in pairs. Thus, we can simply suppose that the number of LTPs is the same as that of RTPs. Note that the number of turning points should be no more than $N_{u}$. Therefore, the length of each array in the algorithm can be set to $N_{u}/2$.

Apparently, the binary search method is the main body of the algorithm, and its computational complexity is $T(L)=O(\log_{2}L)$, where L is the length of the input array. In our algorithm, the maximum value of L is $L=N_{u}/2=2^{n_{s}-1}$, where $n_{s}$ is the resolution of the used DAC. Then we can see that the computational complexity of the algorithm is $O(\log_{2}2^{n_{s}-1})=O(n_{s})$ and the maximum computation time (MCT) of the algorithm corresponds to the case when $L=2^{n_{s}-1}$. In addition to computational complexity, the space complexity of the algorithm is $O(N_{u})=O(2^{n_{s}})$. 

### 2.3. Parameter Identification

**Figure 4:**
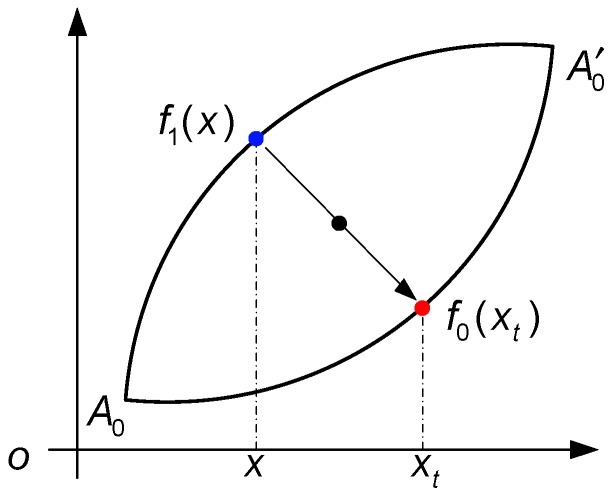
Translation of points on $f_{1}(x)$ to corresponding points on $f_{0}(x)$.

The least mean square method is used to identify the parameters of $f_{0}(x)$ in Equation (9). The oscillating input signals, such as the sinusoidal and triangular waves, between the minimum and maximum input are used to get the input and output data. When the input is increased from the minimum value $x_{A_{0}}$ to the maximum value $x_{A_{0}^{\prime }}$, the trajectory moves along the major ascending curve. However, note that when the input value decreases, the trajectory moves along the descending curve $f_{1}(x)$ rather than $f_{0}(x)$. In order not to lose the information of the descending curve $f_{1}(x)$, the point on the descending curve has to be translated to its corresponding point on the major ascending curve. Note that $f_{0}(x)$ and $f_{1}(x)$ are centrally symmetric, the point $\left(x,f_{1}(x)\right)$ can be translated to its corresponding point $\left(x_{t},f_{0}(x_{t})\right)$, as shown in Figure 4, where

(14) $$\left\{ \begin{array}{l} x_{t}=x_{A_{0}}+x_{A_{0}^{\prime }}-x,\\ f_{0}(x_{t})=f_{0}(x_{A_{0}})+f_{0}(x_{A_{0}^{\prime }})-f_{1}(x). \end{array}\right.$$

**Figure 5:**
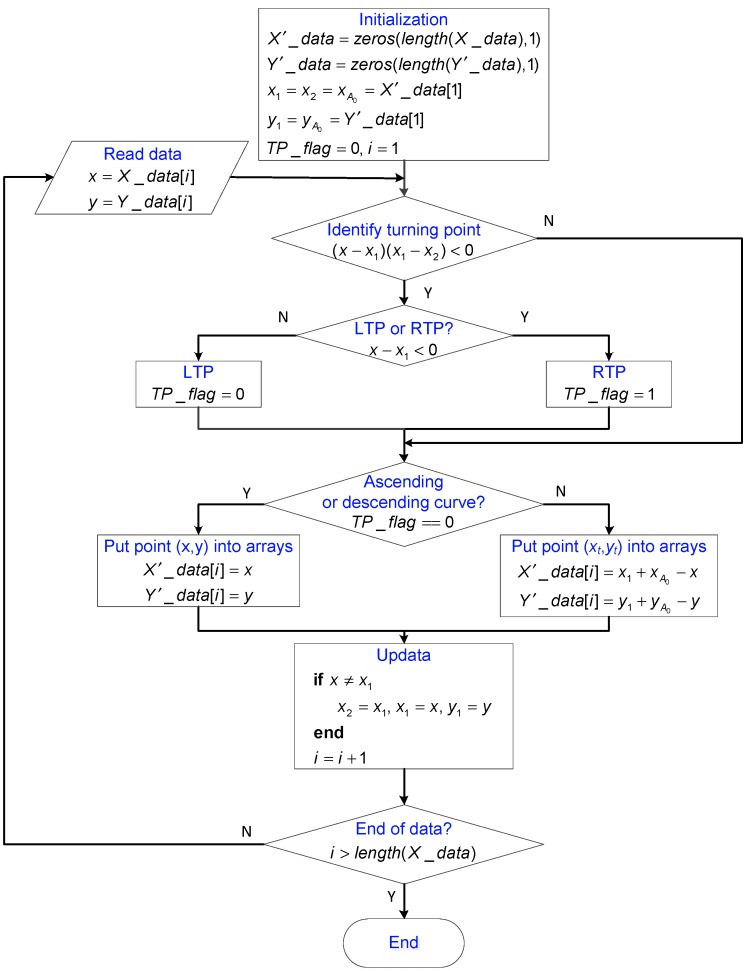
Data reproduction algorithm.

Based on Equation (14), the output–input data can be reproduced by the algorithm shown in Figure 5. In the algorithm, the output–input characteristics of $f_{0}(x)$ can be totally captured by the reproduced arrays $X^{\prime }\_data$ and $Y^{\prime }\_data$. Then the least mean square method can be used to derive the coefficients of $f_{0}(x)$.

**Figure 6:**
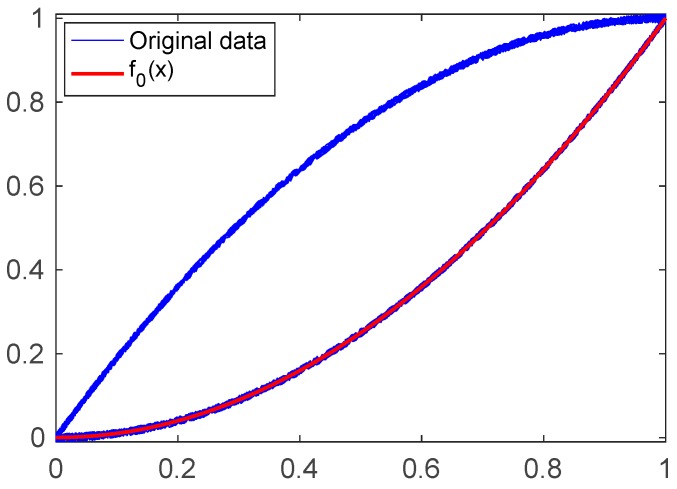
Simulation data and the function $f_{0}(x)$ in Example 1.

Simulation Example 1: We assume that $x^{2}$ (x∈[0, 1]) is the function of the major ascending curve. After the injection of the sinusoidal wave with constant amplitude 1, the input and output data are collected in X_data and Y_data, respectively. The plot of Y_data versus X_data is shown in Figure 6, where the output data are affected by noises. Setting n=2 in Equation (9) and applying the identification algorithm, we have $f_{0}(x)=1.0005x^{2}-0.0008x+0.0002$ (see Figure 6), which is very close to the function $x^{2}$. 

### 2.4. Relationship with the PI Model

Generally, the PI model consists of a set of backlash operators. Considering the fact that input signals for PEAs are positive, one-side backlash operators [20,21] are used in this paper. The PI model with one-side backlash operators can be written as

(15) $$\begin{array}{l} y_{\mathrm{PI}}(0)=\sum_{i=1}^{n_{\mathrm{PI}}}w_{i}H_{i}[x](0)=\sum_{i=1}^{n_{\mathrm{PI}}}w_{i}\max(x(0)-r_{i},\min(x(0),0))\\ y_{\mathrm{PI}}(t)=\sum_{i=1}^{n_{\mathrm{PI}}}w_{i}H_{i}[x](t)=\sum_{i=1}^{n_{\mathrm{PI}}}w_{i}\max(x(t)-r_{i},\min(x(t),y_{\mathrm{PI}}(t-T_{s}))) \end{array}$$

where $H_{i}[x](t)$ is the one-side backlash operator, $w_{i}$ is the weight value, $r_{i}$ is the threshold value, and $n_{\mathrm{PI}}$ is an integer which represents the number of operators. The function of the major ascending curve the PI model is

(16) $$f_{\mathrm{PI}}(x)=\sum_{i=1}^{n_{\mathrm{PI}}}w_{i}\kappa_{i}(x)$$

where

(17) $$\kappa_{i}(x)=\left\{ \begin{array}{l} 0,\;x\in [0,\;r_{i}]\\ x-r_{i},\;x\in (r_{i},\;r] \end{array}\right.$$

It is assumed that $0=r_{1}<r_{2}<\cdots <r_{n_{\mathrm{PI}}}$. At this time, we can present a question as follows: If $f_{\mathrm{PI}}(x)$ is used to replace $f_{0}(x)$, is the trajectory construction method able to compute the output of the PI model? The answer is “yes” since the PI model is symmetric and obedient to Madelung’s rules. In other words, the output of the trajectory construction method is equivalent to that of the PI model. This equivalence is not accidental since the trajectory construction method captures the symmetric property of the PI model and guarantees the equivalence in principle.

**Figure 7:**
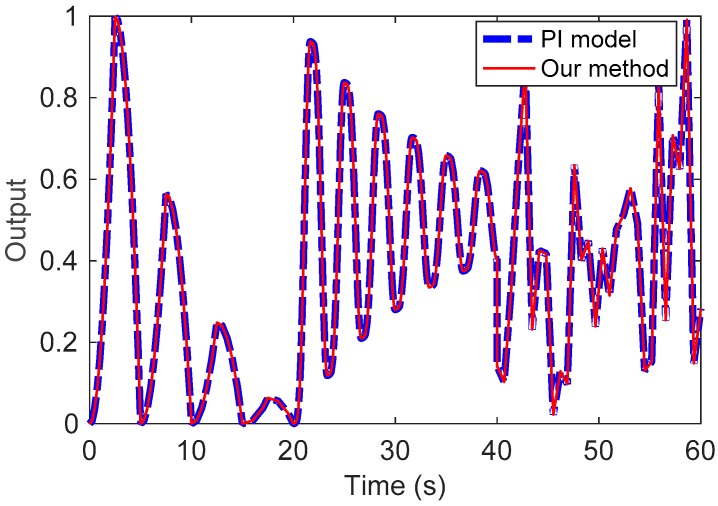
Output signals of the PI model and the presented method.

Simulation Example 2: To simulate the hysteresis phenomenon in Example 1, we use ten operators to construct the PI model. The parameters of the PI model are listed in Table 1. Next, a series of different types of input signals are used to excite both models. The output signals of these two models are also plotted in Figure 7, where we can see that these two methods have the same output signals.

## 3. Simulations and Experiments

### 3.1. HIL Simulations

Hardware-in-the-Loop (HIL) simulations are conducted in this subsection to investigate the computation time of the trajectory construction method with a DSP28335 development board. One pin of the DSP is programmed to denote the state of the algorithm. The pin turns to be a high level when the algorithm starts a new cycle and becomes low at the end of the cycle. The duration of the high level represents the computation time of the trajectory construction method.

**Figure 8:**
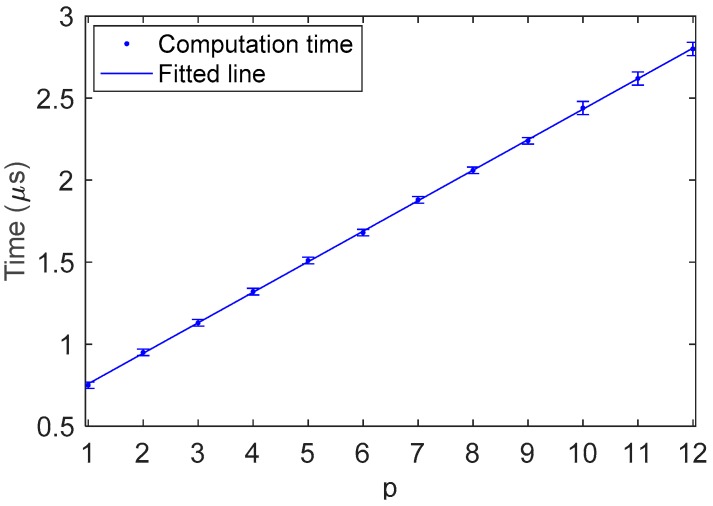
Computation time of the trajectory construction method.

It is obvious from Figure 2 that the computation time depends on the number p of numerical comparisons in the binary search. According to the property of the binary search, there is a logarithmic relation between p and m, with the function given by

(18) $$p=\left\lceil \log_{2}m+1\right\rceil$$

where m is the number of non-empty elements in array $X_{\mathrm{LTP}}$ or array $X_{\mathrm{RTP}}$. It is noted that the computation time increases with the amount of turning points. Fortunately, thanks to the logarithmic relation of Equation (18), p grows slowly with the increase of m. On the other hand, owing to the wiping-out mechanism, m cannot increase infinitely. As mentioned above, the maximum value of m is $N_{u}/2$, where $N_{u}$ represents the number of different input values in the data acquisition system. To cope with the following experiments, we assume $N_{u}{=2}^{n_{s}}=2^{12}=4096$ in the HIL simulations. Figure 8 depicts the relation between computation time and the number of comparisons in the binary search. The data in Figure 8 can be fitted by a straight line 0.19p+0.57, which means that it takes about 0.19 µs to complete a comparison. According to Equation (18), the maximum value of p is

(19) $$p=\left\lceil \log_{2}(N_{u}/2)+1\right\rceil =n_{s}=12$$

and the computation time corresponding to this p value is 2.80 µs, which is also the maximum computation time (MCT). Since the computation time varies with the number of turning points, it is important to estimate the MCT of the trajectory construction method. Furthermore, we can see from Equation (19) that the MCT is related to $n_{s}$, which is the resolution of the DAC.

### 3.2. Experiments

**Figure 9:**
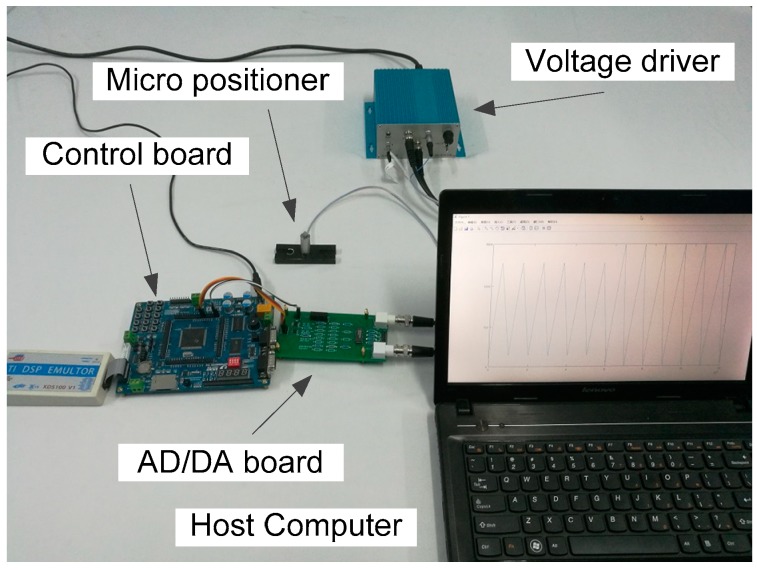
Experimental system.

In this subsection, we will show how to predict the output of PEAs. The experimental system is set up as shown in Figure 9, where the micro positioner 20VS12 actuated by PEA is from Tomorrow Core Company. The positioner has an integrated resistance strain-gauge transducer. The high voltage amplifier XE-650.CA is also from the same company. The data acquisition system consists of the above used DSP28335 development board and an AD/DA board with 12-bit analog-to-digital and digital-to-analog converters. The running frequency of the DSP28335 is set to 150 MHz. The algorithm is compiled on the host computer and then downloaded into the DSP28335.

**Figure 10:**
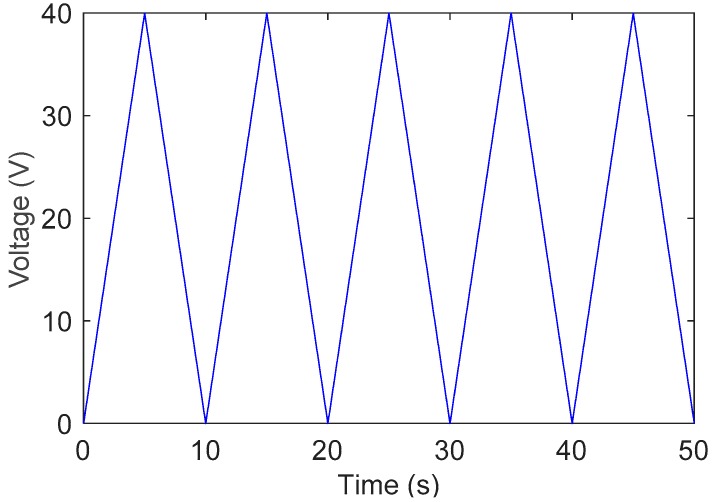
Triangular wave signal.

**Figure 11:**
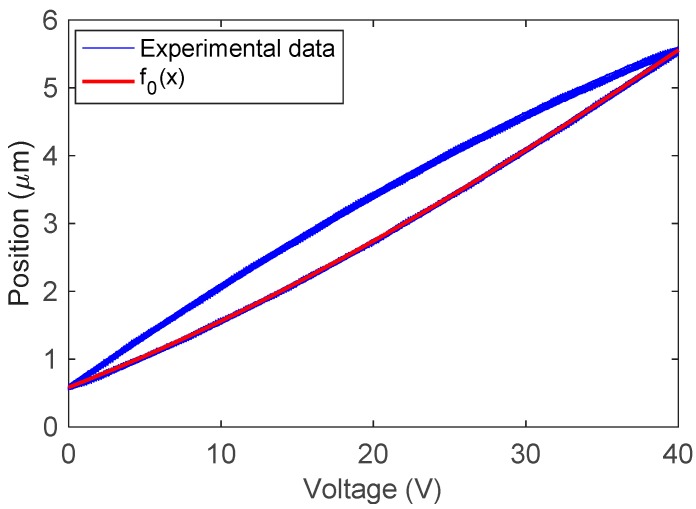
Input and output data used for parameter identification.

First, a triangular wave with constant amplitude 40 V as shown in Figure 10 is used to excite the positioner system to identify the function $f_{0}(x)$. The input–output data are collected and stored in X_data and Y_data, respectively. Figure 11 depicts the plot of Y_data versus X_data. 

Setting n=3 and applying the parameter identification algorithm, we derive the function $f_{0}(x)$ as follows:

(20) $$f_{0}(x)=-6.1876\times 10^{-6}x^{3}+1.1949\times 10^{-3}x^{2}+8.6444\times 10^{-2}x+5.8517\times 10^{-1}$$

**Figure 12:**
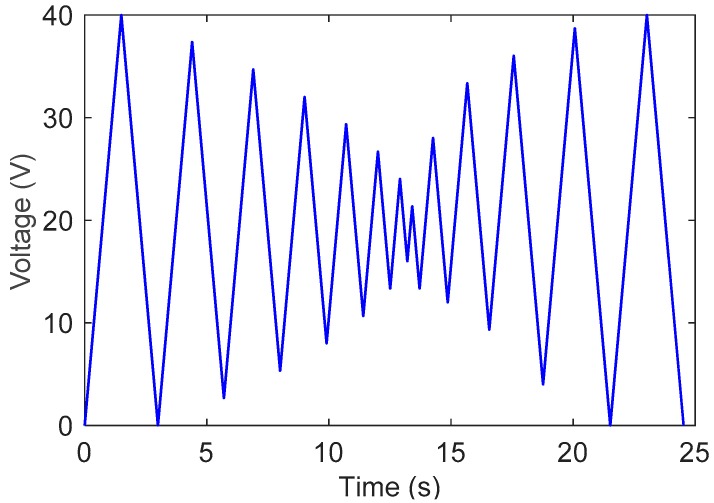
Test signal.

**Figure 13:**
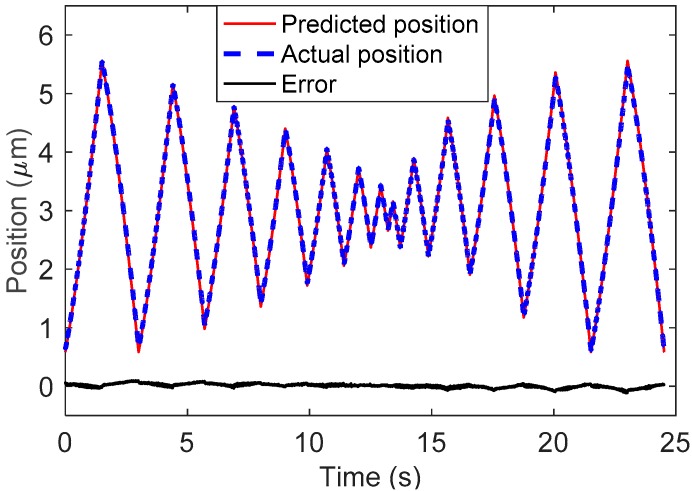
Position prediction results.

The function $f_{0}(x)$ is also plotted in Figure 11, where we can see that the measured data can be well described by $f_{0}(x)$. Next, a test signal is used to validate the effectiveness of the proposed method, as shown in Figure 12. When t∈[0, 13.4], the test signal is a triangular wave with a decreasing amplitude, which begins to increase at t=13.4 s. The position prediction results are shown in Figure 13, where the maximum error is 0.12 µm.

**Figure 14:**
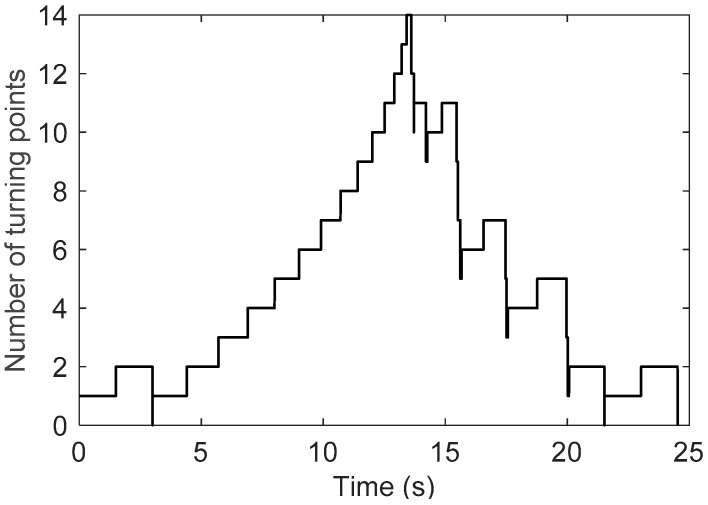
Number of turning points varying with time.

Figure 14 depicts the number of turning points in the above hysteretic movement. The number of turning points increases one at a time and decreases in pairs (an LTP and an RTP, respectively). In the beginning, there is only one turning point, which is the starting point. When the input signal leads back to 0 V at t=3 s, the number of turning points becomes to zero. At t∈[3, 13.4], the number of turning points is monotonically increased. When t>13.4 s, the amplitude of the test signal increases with time. The number of turning points is decreased to zero finally when the test signal arrives at 0 V. As shown in Figure 14, the maximum number of turning points is 14, and more precisely, there are 7 LTPs and 7 RTPs. Thus, we have m=7 and p=4. As shown in Figure 8, the computation time corresponding to p=4 is 1.23 µs. As a matter of fact, the computation time in the whole movement is no greater than 1.23 µs, which is much smaller than the MCT.

### 3.3. Discussions

As shown in Equation (9), there are n+1 parameters if the degree of the polynomial function $f_{0}(x)$ is n. In the experiments, a third order polynomial function (see Equation (20)) is used to describe the major ascending curve. Thus, only four parameters need to be identified. For comparison, if the PI model with ten backlash operators is used, we have to identify twenty parameters. Therefore, the proposed method is superior to the PI model in parameter identification.

However, since the turning points have to be recorded in the proposed method, the worst case has to be considered. From the above analysis, we know that the maximum number of turning points is related to the number of different input values. In other words, a certain amount of memory is needed. However, it is worth pointing out that the demand for memory resources is affordable to most of industrial computers, as shown in Table 2.

Different from traditional operator-based method, the trajectory construction method uses the observed rules for hysteresis (Madelung’s rules) and a measured curve (the major ascending curve) to simulate the symmetric hysteresis. As we have known that hysteresis is a kind of nonlinearity with memory. The concept of turning point is important in the proposed method since turning point is the recorder of movement history. In other words, the turning point is the memory of the hysteretic movement. The proposed method is an intuitive and easy-to-use hysteresis modeling method, and it is also beneficial to the in-depth understanding of the hysteresis phenomenon.

## 4. Conclusions

In this paper, a symmetric hysteresis modeling method has been proposed based on Madelung’s rules. The turning points are recorded and eliminated according to the input value. The key point of the proposed method is to determine the CTP. Then the current can be determined, and the output can be computed by Equation (5). Furthermore, the relationship between our method and the PI model has also been investigated. It is found out that the outputs of both methods are equivalent to each other when $f_{0}(x)$ is replaced with $f_{\mathrm{PI}}(x)$. It should be noted that the proposed method has a relatively high demand for memory resources. Fortunately, the memory consumption is affordable to most of industrial computers. Even embedded processors can be used with low resolution requirements. Simulation and experiment results have shown the effectiveness of the proposed algorithm. In the future work, we will focus on how to reduce the memory usage of the trajectory construction method.

## Figures and Tables

**Table 1:** Parameters of the Prandtl-Ishlinskii (PI) model.

*i*	Weight	Threshold	*i*	Weight	Threshold
1	0.0789	0	6	0.2001	0.5
2	0.2268	0.1	7	0.2000	0.6
3	0.1928	0.2	8	0.2000	0.7
4	0.2019	0.3	9	0.1999	0.8
5	0.1995	0.4	10	0.2005	0.9

**Table 2:** Demand of memory size.

Resolution	12-bit	16-bit	18-bit	24-bit
Memory Size	16 KB	256 KB	1.5 MB	96 MB
